# The Electromagnetic Vacuum Field as an Essential Hidden Ingredient of the Quantum-Mechanical Ontology

**DOI:** 10.3390/e24121717

**Published:** 2022-11-24

**Authors:** Ana Maria Cetto, Luis de la Peña

**Affiliations:** Instituto de Física, Universidad Nacional Autónoma de México, Mexico City 04510, Mexico

**Keywords:** quantum completeness, quantum ontology, zero-point field, quantization mechanism

## Abstract

This paper provides elements in support of the random zero-point radiation field (zpf) as an essential ontological ingredient needed to explain distinctive properties of quantum-mechanical systems. We show that when an otherwise classical particle is connected to the zpf, a drastic, qualitative change in the dynamics takes place, leading eventually to the quantum dynamics. In particular, we demonstrate that in parallel with the evolution of the canonical variables of the particle into quantum operators satisfying the basic commutator $\left[\hat{x},\hat{p}\right]=i\hbar$, also the field canonical variables are transformed, giving rise to the corresponding creation and annihilation operators ${\hat{a}}^{†},\hat{a}$, satisfying $\left[\hat{a},{\hat{a}}^{†}\right]=1$. This allows for an explanation of quantum features such as quantum fluctuations, stationary states and transitions, and establishes a natural contact with (nonrelativistic) quantum electrodynamics.

## 1. Introduction

Occasionally, physicists and philosophers of science express their discomfort with quantum mechanics (qm) by arguing that certain characteristic properties of quantum systems—of atoms in particular—are not correctly taken into account or explained by the usual theoretical narrative, or even appear in open contradiction with the empirical facts. A first example that comes to mind is of course Einstein, whose abiding malaise with quantum theory is well known; other prominent examples are the authors of Refs. [1,2,3,4,5,6]. The pertinence of the criticisms raised cannot be negated, although for the practicing physicist they are normally of marginal importance.

On the other hand, an inspection of the current literature readily reveals the existence of about two dozen different interpretations of qm, some more popular than others, and none of them experimentally verified [7]. How can it be that a fundamental theory that provides the basis for a most significant part of contemporary physics, admits such a variety of alternative, even contradictory interpretations? No serious physicist or philosopher of science in his five senses would claim to come up with a better interpretation of Newtonian mechanics or Maxwellian electrodynamics. Reformulations of a known accepted theory may appear, of course, but fundamental theories do not accept reinterpretations. Special relativity did not reinterpret classical mechanics, it extended mechanics to wider domains, and together with quantum theory helped to specify its range of applicability. We should conclude that in the case of quantum mechanics, such variety of interpretations is indicative of a crucial underdetermination of the theory.

To understand the origin of so many different visions about the same fundamental theory, it is convenient to place ourselves in the context in which qm was born. We recall that the quantum formalism—its excellent mathematical apparatus that we still use today with success—was born in the absence of a deep understanding of the quantum phenomenology. The most important physical guides were Bohr’s 1913 model of the hydrogen atom and the observed atomic spectra with their numerology, explained in part precisely by Bohr’s theory, on one hand, and the discovery by de Broglie of the wave aspects associated with the motion of quantum particles, which almost immediately found empirical verification (alas not explanation) in the form of particle diffraction, on the other.

It is against this background that Heisenberg worked on his version of the theory, the matrix mechanics [8]. Heisenberg discovered that the quantum particles have an unavoidable random behavior. Being persuaded of the completeness of his theory, he took this randomness for an essential, irreducible trait that neither needs nor admits a deeper explanation, and referred to it simply as *quantum fluctuations*. Rather than being received with skepticism by the community, this denomination found extended and immediate acceptance, so much so that even today people continue to speak of quantum fluctuations as an innate phenomenon. An example of how far this conviction has taken ground is that the quantum fluctuations are made responsible for the formation of galaxies in the early stages of the universe—without any proof of their early existence. With his *postulate,* Heisenberg assigned to his nascent theory a noncausal and indeterministic character. Few physicists of the time objected to this view.

A few months after the advent of matrix mechanics, Schrödinger’s wave theory appeared. The new theory helped to get a more complete picture of the quantum world and was well received by many physicists, particularly those who were not close to the leaders of the competing theory. A timely contribution of Schrödinger’s work was that it gave the recipe to construct the matrix elements, which Heisenberg’s mechanics required but had no means to calculate.

On top of this, the description of the atom as an entity that lives in an abstract, mathematical space—a Hilbert space, a well-defined mathematical structure—gives no indication of what is taking place in space-time. A further difficulty is related to the introduction of ’quantum jumps’ between states, which were (and still are, to a large extent) taken as a capricious quantum trait, not amenable to further analysis. Strictly speaking, we rely on a powerful formal description of the atom, with no associated intelligible picture of it.

Unknowingly, Schrödinger’s wave theory implied the introduction of a new element into the quantum description. The point is that electron interference patterns are produced by the accumulation of a high number of point-like events, each one created by a single electron [9]. A single particle produces an isolated, randomly located bright point on the detecting screen, the interference—the wave manifestation—becoming evident only after very many hits. The conclusion—normally one that goes unnoticed—is that Schrödinger’s wave function refers not to a single particle, but to an ensemble of them. Well interpreted, Schrödinger theory is *intrinsically statistical in nature*, and deals with *ensembles* rather than individual particles.

Nevertheless, the statistical perspective of the quantum phenomenon was dismissed in general—and adamantly opposed by the Copenhagen school in particular, which prevails to date under different guises (the statistical description of quantum mechanics was proposed and defended by a few authors, Einstein being the most prominent of them. For a more recent discussion see [10]). This opened the door to another infelicitous ingredient, the observer. The introduction of an *active* character in order to ’explain’ the reduction of the distinctive quantum mixtures to the pure states observed, added a subjective ingredient to the already odd quantum scheme.

All in all, such variety of interpretations and re-interpretations indicates that something of importance is missing in the theory. Having so many variations indicates that the issue is actually not one of interpretation, but of an essential incompleteness. The absence of an appropriate guiding ontological element has turned the physical situation into a mystery. On several occasions people have considered that the solution consists in reshaping the formalism, or adding some hidden variables to the theory, for instance to restore causality, as is the case with Bohmian mechanics [11]. However, rather than the kind of incompletenesses considered by Einstein—any statistical description is incomplete by nature—we are referring to an essential ingredient that is missing. The point is that whatever is to be added to the incomplete theoretical framework should be able to address simultaneously some of its main puzzles, including not just the nature of quantum fluctuations; atomic stability, quantum transitions, discrete atomic spectra, wavelike phenomena and the like should find their natural explanation in a coherent scheme.

## 2. Structure of This Paper

The present paper is one of a series that deals with the development of stochastic electrodynamics (sed) as a physical foundation for quantum mechanics [12,13,14,15,16,17,18,19]. In previous work we have shown that, by including the zero-point radiation field (zpf), sed allows us to arrive at a consistent description of the stationary states and to derive the (nonrelativistic) radiative corrections proper for qed [19,20], in addition to providimg a possible explanation for the so-called quantum jumps [21], along with an understanding of the mechanism of entanglement [22] and of the electron spin as an emergent property [23]. With this paper, we take a step forward by revealing a deeper role for the zpf in relation to quantization. At the outset, in Section 3 we give arguments in favor of the zpf as an element needed to complete the quantum-mechanical ontology. The results presented in the rest of the paper reaffirm these arguments. By revisiting the physical process that leads to the particle’s canonical variables x,p being replaced by the corresponding quantum operators $\tilde{x},\hat{p}$, in Section 4 we show that the permanent action of the background field on an otherwise classical charged particle induces a *qualitative change in the dynamics*. Inspired by this result, in Section 5 we disclose the proces of quantization of the radiation field in interaction with matter and the subsequent emergence of the corresponding creation and annihilation operators ${\hat{a}}^{†},\hat{a}$ satisfying $\left[\hat{a},{\hat{a}}^{†}\right]=1$, thus establishing contact with (nonrelativistic) qed. To illustrate the merits of the present approach, in Section 6 we present the derivation of the (qed) formula for the Einstein coefficient for spontaneous emissions. Some major implications of the results obtained are discussed through the course of the paper. The final section contains a couple of brief concluding remarks.

## 3. The Missing Principle

Because atomic stability and transitions are electromagnetic processes, the missing element must be electromagnetic rather than mechanical in nature. This element should be able to provide at the same time an answer to the questions of quantum causality and the related fluctuations, and of the wavelike behaviour of particles. A natural answer is therefore to consider the stochastic electromagnetic zero-point field (zpf)—the nonthermal component of the radiation field, introduced by Planck in 1912 [24]—as the most suitable candidate (on the origin of this field, a proposal is to consider it as a result of the myriad particles contributing to it along the history of the Universe ([18], Ch. 4). Examples of alternative proposals may be seen in Refs. [25,26]). This proposal is precisely what has been guiding the investigations under the name of *stochastic electrodynamics* (sed). sed was initiated over half a century ago as a result of the physical intuition of the British physicist Trevor Marshall [12], of recent parting. The name itself of the theory contains a hint about the two elements of the prescription, electromagnetic and stochastic. The insertion of the zpf into the picture has the additional virtue of bringing the ensuing theory closer to the successful quantum electrodynamics(qed), as it contains the vacuum fluctuations *ab initio*. Yet contrary to qed, sed considers these fluctuations embodied in a real, random Maxwellian field that fills the entire space with an energy $\left(1/2\right)\hbar \omega$ per normal mode.

Further to providing an explanation for the quantum fluctuations, a most important problem addressed with the introduction of the zpf is the atomic stability—as intuited already at the dawn of quantum mechanics by Nernst [27]. The point is that an atomic electron, like any electric charge in accelerated motion, radiates, thereby losing energy. Yet the stationary atomic states have constant energy. The solution is to consider the existence of a source of energy that can be absorbed by the atoms at the required rate. sed shows this to be the case: when atoms live immersed in the zpf, they may reach a stable state of motion, and they can also make transitions between such states [19,20]. As shown in Section 6, the transitions conventionally dubbed spontaneous are correctly explained by sed, as by qed; not by qm, though, due to its approximate (nonradiative) nature, which entails the neglect of thezpf.

By serving as a bridge that connects the individual particles of a system, this field also serves to generate correlations between their motions even when they do not interact directly, thus giving rise to an apparently nonlocal behavior, as is manifested, for example, in quantum entanglement [22].

Coming back to the conceptual, philosophical considerations, we conclude that the mystery and magic that have accompanied the quantum world over decades may be dissolved by taking the zpfas a *real*, physical field in permanent contact with matter. Considering this field as an inseparable ingredient of the ontology of any quantum system allows us to recover determinism (which in the presence of stochasticity is to be understood as a statistical determinism), causality, locality and a degree of objectivity and realism. *Full* realism, i.e., a description of the dynamics of the atom in space-time, remains a subject for the future.

## 4. Quantization of Matter: The Onset of Operators

### 4.1. The Equation of Motion of Stochastic Electrodynamics

A usual starting point for the analysis of the (nonrelativistic) particle dynamics in sed is the equation of motion, known as the Braffort–Marshall equation, which corresponds to the Abraham–Lorentz equation of electrodynamics in the dipole approximation [12],

(1) $$m\ddot{x}=f\left(x\right)+m\tau \dddot{x}+eE\left(t\right),$$

where f(x) is the external force acting on the particle. As our focus of attention is the atomic problem, we will generally consider a binding force (for example a Coulomb force). The radiation reaction force $m\tau \dddot{x}$, with $\tau =2e^{2}/3mc^{3}$, is the term that leads to the Larmor formula for the power radiated by the accelerated charge. For an electron, $\tau \approx 10^{-23}$ s. E(t) represents the electric component of the zpf taken in the long-wavelength approximation, whose time correlation is given in the continuum limit by (j,k=1,2,3)

(2a) $$\langle E_{k}\left(s\right)E_{j}\left(t\right)\rangle =\delta_{kj}\varphi \left(t-s\right),$$

with the spectral function

$$\varphi \left(t-s\right)=\frac{4\pi }{3}\int_{0}^{\infty }d\omega \;\rho \left(\omega \right)\cos\omega \left(t-s\right).$$

determined by the spectral energy density corresponding to an energy ℏω/2 per mode (notice that this spectral density is Lorentz-invariant and hence is consistent with the law of inertia; see, e.g., [12])

(2b) $$\rho \left(\omega \right)=\frac{\omega^{2}}{\pi^{2}c^{3}}\frac{\hbar \omega }{2},$$

where the first factor is the number of modes per unit volume, whence

(2c) $$\varphi \left(t-s\right)=\frac{2\hbar }{3\pi c^{3}}\int_{0}^{\infty }d\omega \;\omega^{3}\cos\omega \left(t-s\right).$$

Notice that this is the entry point for Planck’s constant, which eventually becomes a measure of the fluctuations in qm.

Following different statistical procedures, it is possible to show that these equations lead to both the Schrödinger and Heisenberg formulations of qm (see [19] and references therein). Here we shall use the same starting point, to address one of the most intriguing traits of quantum theory, namely how it is that the dynamical variables become expressed in terms of operators.

### 4.2. Evolution of the Dynamics: The Field Takes Over

Let us assume that the particle whose motion we want to study, gets connected to the zpf at some instant $t_{o}$. To analyze the effect of the different forces appearing in Equation (1) on the particle dynamics, we introduce an expansion of x(t) in terms of powers of the electric charge *e* [28],

(3) $$x=x^{\left(0\right)}+x^{\left(1\right)}+x^{\left(2\right)}+\ldots =x^{\left(0\right)}+\sum_{s=1}x^{\left(s\right)},$$

where $x_{k}^{\left(s\right)}$ stands for the contribution of order $e^{s}$, *e* being here the coupling factor of the particle to the field. (For neutral electromagnetic particles the coupling would depend on the charge distribution, which may influence the times involved in the evolution of the dynamics.) By inserting (3) in Equation (1) one obtains the hierarchy (summation over repeated indices is understood)

(4) $$m{\ddot{x}}_{i}^{\left(0\right)}=f_{i}\left(x^{\left(0\right)}\right)+m\tau {\dddot{x}}_{i}^{\left(0\right)},$$

(5) $$m{\ddot{x}}_{i}^{\left(1\right)}={\left.\frac{\partial f_{i}}{\partial x_{j}}\right|}_{x^{\left(0\right)}}x_{j}^{\left(1\right)}+m\tau {\dddot{x}}_{i}^{\left(1\right)}+eE_{i}\left(t\right),$$

(6) $$m{\ddot{x}}_{i}^{\left(2\right)}-{\left.\frac{\partial f_{i}}{\partial x_{j}}\right|}_{x^{\left(0\right)}}x_{j}^{\left(2\right)}=\frac{1}{2}{\left.\frac{\partial^{2}f_{i}}{\partial x_{j}\partial x_{k}}\right|}_{x^{\left(0\right)}}x_{j}^{\left(1\right)}x_{k}^{\left(1\right)}+m\tau {\dddot{x}}_{i}^{\left(2\right)},$$

……

In Equation (4) the third-order time derivative may be replaced as customary by the approximate first-order expression τ$\left(df_{i}/dt\right)$. The solution for $x^{\left(0\right)}$ is then seen to decay within a time lapse of the order of the lifetime $\tau_{d}$ determined by the value of $\left|df_{i}/dt\right|$ (an order-of-magnitude calculation of a typical dissipation time gives $\tau_{d}\approx 10^{-11}s$ for a system the size of an atom). This means that the deterministic solution of the homogeneous part of (1) (i.e., in the absence of the zpf) disappears within a time of order $\tau_{d}$, during which the system loses the information of its initial conditions.

By the same argument, the solution of the homogeneous part of Equation (5) also decays after a time of the order of $\tau_{d}$. The solution of the inhomogeneous Equation (5) is a purely stochastic variable, describing a *non-decaying* motion driven by the electric component of the zpf, which may be written in the form

(7) $$x_{i}^{\left(1\right)}=e\int_{-\infty }^{t}dsG_{\mathrm{ik}}\left(t,s\right)E_{k}\left(s\right),$$

where the Green function $G_{\mathrm{ij}}\left(t,s\right)$ is a solution of the equation (to simplify the discussion we omit the term $m\tau {\dddot{x}}_{i}^{\left(1\right)}$, which represents a small correction)

(8) $$m\frac{\partial^{2}}{\partial t^{2}}G_{\mathrm{ik}}\left(t,s\right)-{\left.\frac{\partial f_{i}}{\partial x_{l}}\right|}_{x^{\left(0\right)}}G_{\mathrm{lk}}\left(t,s\right)=\delta \left(t-s\right),$$

with $G_{\mathrm{lk}}\left(t,t\right)=0$ and

(9) $$lim_{s\to t}\frac{\partial }{\partial t}G_{\mathrm{ik}}\left(t,s\right)=\frac{1}{m}\delta_{\mathrm{ik}}.$$

The solution of Equation (8) satisfying these conditions is

(10) $$G_{\mathrm{ik}}\left(t,s\right)={\left.\frac{\partial x_{i}\left(t\right)}{\partial p_{k}\left(s\right)}\right|}_{x^{\left(0\right)}},$$

where the derivative $\partial x_{i}\left(t\right)/\partial p_{k}\left(s\right)$, a function of the exact solution of Equation (1), is to be calculated to zero order in *e*, i.e., at $x^{\left(0\right)}$. The solution of Equation (7) becomes thus

(11a) $$x_{i}^{\left(1\right)}=e\int_{-\infty }^{t}ds{\left.\frac{\partial x_{i}\left(t\right)}{\partial p_{k}\left(s\right)}\right|}_{x^{\left(0\right)}}E_{k}\left(s\right),$$

and its time derivative gives

(11b) $$p_{i}^{\left(1\right)}=e\int_{-\infty }^{t}ds{\left.\frac{\partial p_{i}\left(t\right)}{\partial p_{k}\left(s\right)}\right|}_{x^{\left(0\right)}}E_{k}\left(s\right).$$

These results show that $x_{i}^{\left(1\right)}\left(t\right)$ and $p_{i}^{\left(1\right)}\left(t\right)$ evolve in response to the zpf, at specific rates determined by the external force.

As is clear from (6) and the subsequent equations of the hierarchy, the higher-order solutions $x_{i}^{\left(r\right)}$ are also controlled (although indirectly) by the field, the external force playing now an accessory role. Consequently, along the evolution of the system the dynamics undergoes an *irreversible*, *qualitative* change, from behaving deterministically at $t_{o}$ under the action of the applied force, to eventually acquiring indeterministic properties under the control of the zpf.

### 4.3. Kinematics of the SED System

The observation just made—that the particle dynamics becomes eventually controlled by the background field—calls for an analysis of the evolution of the kinematics used to describe the particle’s response to the field [29]. With this purpose, we set out to calculate the Poisson bracket of the particle’s canonical variables

(12) $${\left\{x_{j}\left(t\right),p_{i}\left(t\right)\right\}}_{xp}=\delta_{ij},$$

with i,j=1,2,3. In this equation, the Poisson bracket is naturally calculated with respect to the variables at time *t*.

The full set of canonical variables includes also those of the infinite number of field modes (a semicolon is used for the set of canonical variables, to avoid confusion with the Poisson bracket),

(13) $$\left\{q;p\right\}=\left\{x_{i},q_{\alpha };p_{i},p_{\alpha }\right\},$$

with $q_{\alpha },p_{\alpha }$ the canonical variables corresponding to the mode of the radiation field of (angular) frequency $\omega_{\alpha }$. (A discrete set of frequencies is considered here, for reasons that will become clear later.) At the initial time $t_{o}$, when particle and field start to interact, the full set of canonical variables is given by

(14) $$\left\{q_{o};p_{o}\right\}=\left\{x_{io},q_{\alpha o};p_{io},p_{\alpha o}\right\},$$

with $x_{io},p_{io}$ the initial values of the particle’s variables, and $q_{\alpha o},p_{\alpha o}$ corresponding to the original field modes, which are those of the zpf alone. Because the system as a whole is Hamiltonian, the set at any time *t*, $\left\{q;p\right\}$, is related to the set $\left\{q_{o};p_{o}\right\}$ via a canonical transformation, and therefore the Poisson bracket of any two functions f,g can be taken with respect to either of them,

(15) $${\left\{f,g\right\}}_{qp}={\left\{f,g\right\}}_{q_{o}p_{o}}$$

$$={\left\{f,g\right\}}_{x_{o}p_{o}}+{\left\{f,g\right\}}_{q_{\alpha o}p_{\alpha o}}.$$

At any time the particle variables $x_{i},p_{j}$ satisfy the condition

(16) $${\left\{x_{i}\left(t\right),p_{j}\left(t\right)\right\}}_{qp}=\delta_{ij},$$

whence according to Equation (15),

(17) $${\left\{x_{i}\left(t\right),p_{j}\left(t\right)\right\}}_{x_{o}p_{o}}+{\left\{x_{i}\left(t\right),p_{j}\left(t\right)\right\}}_{q_{\alpha o}p_{\alpha o}}=\delta_{ij}.$$

As noted earlier in connection with Equation (4), due to the radiation reaction the particle loses track of its initial conditions $x_{o},p_{o}$, after a time of the order of $\tau_{d}$, so the first term in Equation (17) vanishes,

(18) $${\left\{x_{i}\left(t\right),p_{j}\left(t\right)\right\}}_{q_{o}p_{o}\;\vec{t>\tau_{d}}}{\left\{x_{i}\left(t\right),p_{j}\left(t\right)\right\}}_{q_{\alpha o}p_{\alpha o}}.$$

This means that eventually, the particle kinematics becomes defined by the canonical variables of the field modes with which it interacts,

(19) $${\left\{x_{i}\left(t\right),p_{j}\left(t\right)\right\}}_{q_{\alpha o}p_{\alpha o}}=\delta_{ij}\;\left(t>\tau_{d}\right).$$

Using the rules of transformation from canonical to normal variables,

(20) $$\omega_{\alpha }q_{\alpha }^{o}=\sqrt{\hbar \omega_{\alpha }/2}\left(a_{\alpha }+a_{\alpha }^{*}\right),\;p_{\alpha }^{o}=-i\sqrt{\hbar \omega_{\alpha }/2}\left(a_{\alpha }-a_{\alpha }^{*}\right),$$

as corresponds to the zpf, we calculate the transformed Poisson bracket with respect to the new variables. We define the bilinear form $\left[f,g\right]$ in general as this transformed Poisson bracket,

(21) $$\sum_{\alpha }\left({\frac{\partial f}{\partial a}}_{\alpha }\frac{\partial g}{\partial a_{\alpha }^{*}}-{\frac{\partial g}{\partial a}}_{\alpha }\frac{\partial f}{\partial a_{\alpha }^{*}}\right)=i\hbar {\left\{f,g\right\}}_{q_{\alpha o}p_{\alpha o}}\equiv \left[f,g\right].$$

Applied to the canonical particle variables, this gives

(22) $$\left[x_{i},p_{j}\right]=i\hbar {\left\{x_{i},p_{j}\right\}}_{q_{\alpha o}p_{\alpha o}}.$$

According to Equation (19), for times $t>\tau_{d}$ this bilinear form satisfies the condition

(23) $$\left[x_{i},p_{j}\right]=i\hbar \delta_{ij}.\;\left(t>\tau_{d}\right)$$

This is a noteworthy result: it indicates that the symplectic relation between the particle variables $x_{i}$ and $p_{j}$ becomes eventually determined by their functional dependence on the normal zpf variables, with the scale given by Planck’s constant. In the following section, we discuss this drastic change in more detail.

### 4.4. Disclosing the Origin of the Quantum Operators

As mentioned earlier, by including the zpf, sed provides for the existence of stationary states [19]. Given its universal character, Equation (23) may be applied to any state *n*, be it the ground state or an excited state. We may therefore tag the variables $x_{i},p_{j}$ with the subindex *n* and write, using (21),

(24) $${\left[x_{i},p_{j}\right]}_{nn}=\sum_{\alpha }\left(\frac{\partial x_{n}}{\partial a_{\alpha }}\frac{\partial p_{n}}{\partial a_{\alpha }^{*}}-\frac{\partial p_{n}}{\partial a_{\alpha }}\frac{\partial x_{n}}{\partial a_{\alpha }^{*}}\right)=i\hbar \delta_{ij}.$$

In what follows, we limit the discussion to the one-dimensional case, for simplicity. The constant value of this bilinear form implies that the variables $x_{n}$ and $p_{n}=m{\dot{x}}_{n}$ are linear functions of the set $\left\{a_{\alpha },a_{\alpha }^{*}\right\}$. The field modes involved are those to which the particle responds, namely those that can take the particle from state *n* to another state, say k. We therefore write [29] (see also [18], Ch. 10),

(25) $$x_{n}\left(t\right)=\sum_{k}x_{nk}a_{nk}e^{-i\omega_{kn}t}+c.c.,\;\;p_{n}\left(t\right)=\sum_{k}p_{nk}a_{nk}e^{-i\omega_{kn}t}+c.c.,$$

where $a_{nk}$ is the normal variable associated with the field mode that connects state *n* with state *k*, and $x_{nk}$, $p_{nk}=-im\omega_{kn}x_{nk}$ are the respective response coefficients. Introduction of these expressions into Equation (24) gives

$${\left[x,p\right]}_{nn}=2im\sum_{k}\omega_{kn}{\left|x_{nk}\right|}^{2}=i\hbar ,$$

where we recognize the well-known Thomas–Reiche–Kuhn sum rule,

(26) $$\sum_{k}\omega_{kn}{\left|x_{nk}\right|}^{2}=\hbar /2m.$$

Further, since the field variables $a_{nk},a_{n^{\prime }k}$ connecting different states $n,n^{\prime }$ with state *k* are independent random variables, by combining Equations (21), (25) and (26) one obtains

(27) $${\left[x,p\right]}_{nn^{\prime }}=i\hbar \delta_{nn^{\prime }}.$$

The quantities $x_{nk}$ and $a_{nk}$ refer to the transition n→k involving the frequency $\omega_{kn}$, whilst $x_{kn}$ and $a_{kn}$ refer to the inverse transition, with $\omega_{nk}=-\omega_{kn}$; therefore, from (25), $x_{nk}^{*}\left(\omega_{nk}\right)=x_{kn}\left(\omega_{kn}\right),\;p_{nk}^{*}\left(\omega_{nk}\right)=p_{kn}\left(\omega_{kn}\right),\;a_{nk}^{*}\left(\omega_{nk}\right)=a_{kn}\left(\omega_{kn}\right),$ whence Equation (27) takes the form

(28) $$\sum_{k}\left(x_{nk}p_{kn^{\prime }}-p_{n^{\prime }k}x_{kn}\right)=i\hbar \delta_{nn´}.$$

Given the structure of this equation, the coefficients $x_{nk}$ and $p_{nk}$ can be organized as the elements of matrices $\hat{x}$ and $\hat{p}$, respectively, with as many rows and columns as there are different states, so that (28) becomes

(29) $${\left[\hat{x},\hat{p}\right]}_{nn^{\prime }}=i\hbar \delta_{nn^{\prime }},$$

which is the matrix formula for the quantum commutator

(30) $$\left[\hat{x},\hat{p}\right]=i\hbar .$$

We see that as a result of the evolution of the kinematics, the Poisson bracket of the particle’s canonical variables x,p has turned into the quantum commutator. The relationship between Poisson brackets and commutators established with great insight by Dirac in the early days of qm finds herewith a possible physical explanation.

The rest of the formalism is obtained by introducing the vectors representing the stationary states of the particle on which the operators act, denoted by $|n\rangle$. From this point on, the Hilbert-space formulation may be completed following the standard procedure [29].

Seen as a component of the operator $\hat{x}$ acting on the system, $x_{nk}$ represents the response to the field mode (nk) that takes the system from state *n* to state *k* and vice versa, as expressed by the selection rules for dipolar transitions. An application of these selection rules is illustrated in Section 6, where the Einstein coefficient corresponding to a (spontaneous) transition n→k is shown to depend directly on ${\left|x_{nk}\right|}^{2}$.

It is clear from the above that the operators $\hat{x},\hat{p}$ do not describe particle trajectories, and no phase-space description is associated with them. The Hilbert-space formalism represents a compact and elegant way of describing the quantum behavior based on the transitions between states, at the cost of a space-time description of what happens inside the atom.

This result offers a response to a long-standing, intriguing question of qm that sounds like an oxymoron: how is it possible that the description of a *stationary* quantum state is made in terms of a collection of *transition* amplitudes between states? The suggested functionality of the transition coefficients as the building blocks of the operator representation has moreover an historical value, since in the hands of Born (and as was unknowingly suggested by Heisenberg) they became the omnipresent elements of the quantum description.

## 5. Quantization of the Field

### 5.1. Describing a Field Mode in Interaction with Matter

We now proceed to develop a description of the effect produced on the radiation field by its interaction with matter, consistent with the above. This means that rather than assuming that the (free) radiation field is quantized ab initio, or postulating the canonical quantization of the field by simple analogy, we shift the focus to that part of the field that is exchanging energy with quantized matter in order to understand how the field quantization comes about. The description to be developed should serve to express any electric or magnetic component of the interacting field, be it the zpf alone or in combination with an external field.

Initially, when particle and field became coupled, both particle and field were ’free’ and the entire spectrum of field modes was in principle involved in the interaction (see, however, the discussion in Section 5.5). However, once matter has become quantized as described above, the field modes in interaction with it belong to the reduced set $\left\{\alpha \right\}=\left\{nk\right\}$.

The fact that quantized matter interacts selectively with single field modes of well-defined frequencies allows us to focus on one such mode, which amounts to working with a single Fourier component of the field, having a momentum k with $\left|k\right|=\omega /c$, polarization ϵ and angular frequency $\omega =\left|\omega_{nk}\right|$. The electric and magnetic Fourier components corresponding to this mode can be expressed, as usual, in terms of the respective canonical variables q(t),p(t) (see, e.g., [30,31]).

Let us assume that the field mode of frequency ω is in a given state, which we call n. We denote with $q_{n}\left(t\right)$, $p_{n}\left(t\right)$ the canonical variables of this mode. and express these in terms of a set of normal field variables $a_{{\mathrm{nn}}^{\prime }}$, with respective coefficients $q_{{\mathrm{nn}}^{\prime }}$, $p_{{\mathrm{nn}}^{\prime }}$:

(31) $$q_{n}\left(t\right)=\sum_{n^{\prime }}q_{{\mathrm{nn}}^{\prime }}a_{{\mathrm{nn}}^{\prime }}e^{-i\omega_{n^{\prime }n}t}+c.c.,\;p_{n}\left(t\right)=\sum_{n^{\prime }}p_{{\mathrm{nn}}^{\prime }}a_{{\mathrm{nn}}^{\prime }}e^{-i\omega_{n^{\prime }n}t}+c.c.,$$

where

(32) $$p_{{\mathrm{nn}}^{\prime }}=-i\omega_{n^{\prime }n}q_{{\mathrm{nn}}^{\prime }},$$

and $n^{\prime }$ denotes the states of the field mode that can be reached from state n as a result of its interaction with matter. Since the field mode has a single well-defined frequency ω, necessarily $\left|\omega_{n^{\prime }n}\right|=\omega$, i.e., $\omega_{n^{\prime }n}=\pm \omega$. Consequently, only two of the coefficients $q_{{\mathrm{nn}}^{\prime }}$ connecting state n with some other state $n^{\prime }$ are different from zero. Since there are no intermediate states, we may identify the immediately upper state of the field (corresponding to $\omega_{n^{\prime }n}=\omega$) with $n^{\prime }=n\;+\;1$, and the immediately lower state (corresponding to $\omega_{n^{\prime }n}=-\omega$) with $n^{\prime }=n\;-\;1$, so that Equation (31) becomes

(33) $$q_{n}\left(t\right)=q_{\mathrm{nn}+1}a_{\mathrm{nn}+1}e^{-i\omega t}+q_{\mathrm{nn}-1}a_{\mathrm{nn}-1}e^{i\omega t}+c.c.,$$

(34) $$p_{n}\left(t\right)=-i\omega q_{\mathrm{nn}+1}a_{\mathrm{nn}+1}e^{-i\omega t}+i\omega q_{\mathrm{nn}-1}a_{\mathrm{nn}-1}e^{i\omega t}+c.c,$$

and because of (32),

(35) $$\omega q_{\mathrm{nn}+1}-ip_{\mathrm{nn}+1}=0,\;\omega q_{\mathrm{nn}-1}+ip_{\mathrm{nn}-1}=0.$$

In analogy with Equations (25), we identify $q_{\mathrm{nn}+1},q_{\mathrm{nn}-1}$ with the *active* (or *response*) *coefficients* involved in the change of state of the field in interaction with matter, from n to n+1 and n−1, respectively.

### 5.2. Genesis of the Field Operators

Let us now take the Poisson bracket of the canonical field variables pertaining to the single field mode,

(36) $$\left\{q_{n}\left(t\right),p_{n}\left(t\right)\right\}=1,$$

and calculate it with respect to the complete set of canonical variables at the initial time $t_{0}$. By the same argument used in Section 4.3, after a time of order $\tau_{d}$, when the system has lost track of the initial values of the particle variables,

(37) $${\left\{q_{n}\left(t\right),p_{n}\left(t\right)\right\}}_{q_{o}p_{o}\;\vec{t>\tau_{d}}}{\left\{q_{n}\left(t\right),p_{n}\left(t\right)\right\}}_{q_{\alpha o}p_{\alpha o}}.$$

only the Poisson bracket with respect to the original zpf quadratures $q_{\alpha o},p_{\alpha o}\;$ survives,

(38) $${\left\{q_{n}\left(t\right),p_{n}\left(t\right)\right\}}_{q_{\alpha o}p_{\alpha o}}=1.$$

The similarity transformation from canonical zpf quadratures to normal field variables $a_{\alpha },a_{\alpha }^{*}$, Equations (20), leads to the transformed Poisson bracket,

(39) $$\left[q_{n}\left(t\right),p_{n}\left(t\right)\right]=i\hbar .$$

On account of (33) and (34), $q_{n}\left(t\right)$ and $p_{n}\left(t\right)$ depend only on the normal variables $a_{\mathrm{nn}\pm 1}$, and Equation (39) gives therefore

(40) $$\sum_{n^{\prime }=n\pm 1}\left(q_{{\mathrm{nn}}^{\prime }}p_{{\mathrm{nn}}^{\prime }}^{*}-p_{{\mathrm{nn}}^{\prime }}q_{{\mathrm{nn}}^{\prime }}^{*}\right)=i\hbar .$$

By applying Equation (33) successively to n and to n’, we note that

(41) $$q_{{\mathrm{nn}}^{\prime }}^{*}\left(\omega_{n^{\prime }n}\right)=q_{n^{\prime }n}\left(\omega_{{\mathrm{nn}}^{\prime }}\right),\;p_{{\mathrm{nn}}^{\prime }}^{*}\left(\omega_{n^{\prime }n}\right)=p_{n^{\prime }n}\left(\omega_{{\mathrm{nn}}^{\prime }}\right),\;a_{{\mathrm{nn}}^{\prime }}^{*}\left(\omega_{n^{\prime }n}\right)=a_{n^{\prime }n}\left(\omega_{{\mathrm{nn}}^{\prime }}\right),$$

so that Equation (40) can be written in the alternative form

(42) $$\sum_{n^{\prime }=n\pm 1}\left(q_{{\mathrm{nn}}^{\prime }}p_{n^{\prime }n}-p_{{\mathrm{nn}}^{\prime }}q_{n^{\prime }n}\right)=i\hbar .$$

By identifying $q_{{\mathrm{nn}}^{\prime }}$ and $p_{n^{\prime }n}$ as the elements of matrices $\hat{q}$ and $\hat{p}$, respectively, Equation (42) becomes

(43) $$\left[\hat{q},\hat{p}\right]=i\hbar ,$$

for any state n of the field.

Equation (43) tells us that after a time lapse of order $\tau_{{}_{d}}$, the (transformed) Poisson bracket of the canonical field variables q(t),p(t) has evolved towards the canonical field commutator. The matrix elements of $\hat{q},\hat{p}$ represent the transition coefficients (or ’amplitudes’) between field states. Notice that, once more, the Hamiltonian nature of the entire system ensures the preservation of the symplectic structure in the process of transition from the initial Poisson bracket to the final commutator.

As just stated, the matrix elements of $\hat{q},\hat{p}$ determine the possible changes of state of the field mode in interaction with matter. Since $n^{\prime }=n\pm 1$, $\hat{q}$ and $\hat{p}$ have off-diagonal elements immediately above and below the diagonal only. Further, from (32) we have $p_{{\mathrm{nn}}^{\prime }}+i\omega_{n^{\prime }n}q_{{\mathrm{nn}}^{\prime }}=0$. Therefore, the normalized matrix $\hat{a}$ and its adjoint, defined as

(44) $$\hat{a}=\frac{1}{\sqrt{2\hbar \omega }}\left(\omega \hat{q}+i\hat{p}\right),\;\;{\hat{a}}^{†}=\frac{1}{\sqrt{2\hbar \omega }}\left(\omega \hat{q}-i\hat{p}\right),$$

have offdiagonal elements either immediately above or immediately below the diagonal, meaning that they play the role of annihilation and creation operators, respectively. In terms of these, (43) becomes

(45) $$\left[\hat{a},{\hat{a}}^{†}\right]=1.$$

We recall that the canonical variables $q_{n}\left(t\right),p_{n}\left(t\right)$, (33) and (34), describe a field mode of frequency ω with given momentum and polarization $\left(k,\epsilon \right)$. Since the normal variables $a_{nn^{\prime }}$ pertaining to different field modes have independent random phases, we may generalize Equation (45) to operators ${\hat{a}}_{k\epsilon },{\hat{a}}_{k^{\prime }\epsilon^{\prime }}$, and write

(46) $$\left[{\hat{a}}_{k\epsilon },{\hat{a}}_{k^{\prime }\epsilon^{\prime }}^{†}\right]=\delta_{{\mathrm{kk}}^{\prime }}^{3}\delta_{\epsilon \epsilon^{\prime }}.$$

The rest of the quantum formalism is obtained by introducing the vectors representing the possible states of the field mode on which the operators act, which are denoted by $|n\rangle$, with $|0\rangle$ for the ground state, $\hat{a}|0\rangle =0$. The result of operating with $\hat{a}$ or ${\hat{a}}^{†}$ on state $|n\rangle$ is obtained by applying (45) iteratively, as usual:

(47) $$\hat{a}|n\rangle =\sqrt{n}|n-1\rangle ,\;{\hat{a}}^{†}|n\rangle =\sqrt{n+1}|n+1\rangle .$$

### 5.3. On the Meaning of the Quantum Field Operators

The results obtained above are in agreement with the established fact that light and matter in interaction exchange radiant energy in well-defined quantities, ±ℏω. By writing the Hamiltonian operator associated with a single mode as

(48) $$\hat{H}=\frac{1}{2}\left(\omega^{2}{\hat{q}}^{2}+{\hat{p}}^{2}\right)=\frac{\hbar \omega }{2}\left(\hat{a}{\hat{a}}^{†}+{\hat{a}}^{†}\hat{a}\right),$$

and using (47), one obtains the well-known expression for the energy expectation value

(49) $$\langle n|\hat{H}|n\rangle =E_{n}=\left(n+\frac{1}{2}\right)\hbar \omega ,$$

whence indeed $\left|E_{n}-E_{n\pm 1}\right|=\hbar \omega$.

Given that the effect of the operators $\hat{a},{\hat{a}}^{†}$ is to lower or raise the number n, respectively, it seems appropriate to identify the separate expressions

(50) $${\hat{H}}^{a}=\hbar \omega {\hat{a}}^{†}\hat{a},\;{\hat{H}}^{e}=\hbar \omega \hat{a}{\hat{a}}^{†},$$

with the operators corresponding to the field energy available for processes involving either absorption or emission of radiation by matter, respectively, according to the order of the individual operators acting on state $|n\rangle$,

(51) $$\langle n|{\hat{H}}^{a}|n\rangle =n\hbar \omega ,\;\;\langle n|{\hat{H}}^{e}|n\rangle =\left(n+1\right)\hbar \omega .$$

The operators $\hat{a},{\hat{a}}^{†}$ are sometimes called the *fictitious harmonic oscillators* associated with a given field mode (see, e.g., [30]). However, the electric and magnetic components of a field mode are real physical entities; they are described in terms of the canonical variables $q_{n}\left(t\right),p_{n}\left(t\right)$. In their turn, the operators $\hat{q},\hat{p}$ (or $\hat{a},{\hat{a}}^{†}$) represent the response of a field mode to its interaction with quantized matter, which may lead to a change of state of the field with the concomitant loss, viz gain, of an energy ℏω.

### 5.4. On the Quantization of the Free Electromagnetic Field

It is commonly accepted that the description of the radiation field in terms of operators applies in principle to modes of all frequencies, and that it is only for practical reasons that a distinction may be convenient when dealing with different parts of the spectrum. As argued by Mandel and Wolf [31], “in the microwave region of the spectrum, and still at longer wavelenghts, the number of photons is usually very large, and we are justified in treating the system classically”.

This prevalent view stands in contrast with the conclusions derived from the present work. Equations (48)–(51), as all the previous ones, refer to a field mode that exchanges energy *as a result of its interaction with quantized matter*. Consequently, they do not tell us much about the (quantum or non-quantum) nature of the *free* radiation field, or the field emitted or detected by other means. It is certainly tempting to use the elegant quantum formalism to describe any radiation field of whatever frequency. After all, the quantum theory of radiation has gained a special status as “the most successful and embracing theory of optics” [31]. However the conclusions derived from it may not necessarily be applicable beyond the optical region, or more generally, in cases in which the field is emitted or detected by a nonquantized source. What is the evidence that low-frequency waves are quantized? Take for example a beam (or pulse) of radio waves produced by the oscillating elements of a transmitting antenna, or the radiation emitted by a beam of freely moving, accelerated charges, which in principle can have a continuous range of energies. We can only safely say that the radiation is quantized when it is produced (or absorbed) as a result of a transition between quantum states of matter.

### 5.5. A Note on the Reality of the Vacuum Field

In quantum theory, the vacuum (or vacuum fluctuations) is conventionally considered as fictitious, or as a virtual entity created spontaneously by particle–antiparticle pairs. Without this notion conditioning the validity of the results presented here, we favor the perspective of the vacuum as a real Maxwellian electromagnetic field in its ground state. Precisely because it represents the ground state of the complete radiation field, no energy can be effectively extracted from it by means of atomic transitions, as indicated in (51) with n=0; hence, it does not activate optical detectors, and no *direct* spectroscopic evidence is available to prove its existence. The fact that it is not part of the photonic field, however, neither invalidates its reality nor diminishes the consequences of its permanent action on matter, as illustrated in the present work. A number of other important results widely reported in the literature, including the Casimir effect [32], the Van der Waals forces [14,33], diamagnetism [14,34] and the Lamb shift ([32,35] and [19] ch. 6), not to mention the emergence of characteristic quantum phenomena such as entanglement and atomic stability [19], speak to the inescapable reality of this field.

As observed by Milonni and Smith [36], the zero-point contributions are essential to their derivation of the van der Waals interaction. The vacuum fluctuations are introduced in [36], as is normally done, in passing from a classical description of the field to a quantized one—in contrast to sed, where they are present from the start. Ref. [37] contains further examples of effects that stem from the electromagnetic zero-point fluctuations within a quantum framework. From the usual qed perspective, as mentioned in [37], the sed use of a real zpf—considered as a semiclassical approximation—has merely the advantage of physical transparency. Nevertheless, the effects produced by the zero-point fluctuations are unquestionably real.

This automatically leads us to the problem of the infinite energy content of the zpf, when the spectral energy density given by Equation (2b) is integrated over the entire spectrum,

(52) $$E=\int_{0}^{\infty }d\omega \;\rho \left(\omega \right)=\frac{\hbar }{2\pi^{2}c^{3}}\int_{0}^{\infty }d\omega \;\omega^{3}.$$

It is not our intention to address here this well-known and not yet resolved problem that has beset modern physics (see, e.g., [4]), and for which alternative solutions have been advanced (e.g., [38]). Nevertheless, in the light of the perspective gained with the work on stochastic electrodynamics reported in Refs. [12,13,14,15,16,17,18,19] and the results we have presented here, it is clear that at least at the level of the present description, Equation (52) poses no problem. The capacity of massive particles to respond to the radiation field is limited by their inertia, meaning that they become transparent and are unable to respond to infinitely high-frequency field modes and, for the same reason, they are unable to radiate at such high frequencies. Further, when already in the quantum regine, atomic matter interacts with field modes of frequencies lying within a limited range, as discussed above, and may be considered to be transparent to the rest of the spectrum. In addition, the cutoff frequency (of the order of Compton’s frequency $mc^{2}/\hbar$) frequently introduced in qed and leading to numerically correct results for the radiative corrections, signals the existence of a physical limit to the frequencies at which particles and field modes exchange energy, beyond which other kinds of phenomena take place. Do physical phenomena still occur at even higher frequencies? Where does the radiation spectrum end? These are still open questions, to which cosmologists and high-energy physicists may eventually find an answer.

## 6. Energy Balance: Atomic Stability and Contact with QED

Let us apply the results presented here to a problem of interest, to get a feeling for the kind of conclusions that may be drawn from them. We are interested in knowing what happens with the average energy of the particle once its response has become controlled by the field. We shall assume that the particle is in a stationary state *n*, which may be the ground state or an excited state. The equation of evolution for its average energy is obtained by multiplying the dynamical Equation (1) with p and averaging over the ensemble [20,28],

(53) $$\frac{d}{dt}{\langle H\rangle }_{n}=\tau {\langle p\cdot \dddot{x}\rangle }_{n}+\frac{e}{m}{\langle p\cdot E(t)\rangle }_{n}.$$

The first term on the r.h.s. represents the average power lost by radiation reaction, and the second term represents the average power absorbed by the particle from the field.

For an explicit calculation of these terms, we use the results of Section 4. Specifically, we observe that in Equation (11) the arguments under the integral contain factors of the form $\partial x_{i}\left(t\right)/\partial p_{j}\left(s\right)$, $\partial p_{i}\left(t\right)/\partial p_{j}\left(s\right)$, which may be written in terms of Poisson brackets,

(54) $$\partial x_{i}\left(t\right)/\partial p_{k}\left(s\right)={\left\{x_{k}\left(s\right),x_{i}\left(t\right)\right\}}_{xp},\;\partial p_{i}\left(t\right)/\partial p_{k}\left(s\right)={\left\{x_{k}\left(s\right),p_{i}\left(t\right)\right\}}_{xp},$$

According to what we have learned, for times s,t larger than $\tau_{d}$ these Poisson brackets should be replaced by the corresponding quantum commutators,

(55) $${\left\{x_{k}\left(s\right),x_{i}\left(t\right)\right\}}_{xp}\to \frac{1}{i\hbar }\left[{\hat{x}}_{k}\left(s\right),{\hat{x}}_{i}\left(t\right)\right],\;{\left\{x_{k}\left(s\right),p_{i}\left(t\right)\right\}}_{xp}\to \frac{1}{i\hbar }\left[{\hat{x}}_{k}\left(s\right),{\hat{p}}_{i}\left(t\right)\right].$$

By setting $t_{o}=-\infty$, the initial contribution to the integral for $t_{o}\leq s\leq t_{o}+\tau_{d}$ may be safely neglected, and Equation (11) take therefore the form

(56a) $$x_{i}^{\left(1\right)}=\frac{e}{i\hbar }\int_{-\infty }^{t}ds\left[{\hat{x}}_{k}\left(s\right),{\hat{x}}_{i}\left(t\right)\right]E_{k}\left(s\right),$$

(56b) $$p_{i}^{\left(1\right)}=\frac{e}{i\hbar }\int_{-\infty }^{t}ds\left[{\hat{x}}_{k}\left(s\right),{\hat{p}}_{i}\left(t\right)\right]E_{k}\left(s\right).$$

Writing the commutator in terms of the matrix elements corresponding to state *n* with the help of Equations (25) and (26) gives

(57) $${\left[{\hat{x}}_{i}\left(s\right),\hat{p_{i}}\left(t\right)\right]}_{nn}=2im\sum_{i}\sum_{k}{\left|x_{ink}\right|}^{2}\omega_{ikn}\cos\omega_{ikn}\left(t-s\right),$$

whence from Equations (2) and (56b),

$$e{\langle p\cdot E\rangle }_{n}=$$

$$=\frac{4me^{2}}{3\pi c^{3}}\sum_{i}\sum_{k}{\left|x_{ink}\right|}^{2}\omega_{ikn}\int_{0}^{\infty }d\omega \;\omega^{3}\int_{-\infty }^{t}ds\cos\omega \left(t-s\right)\cos\omega_{ikn}\left(t-s\right)$$

(58) $$=\frac{2me^{2}}{3c^{3}}\sum_{i}\sum_{k}{\left|x_{ink}\right|}^{2}\omega_{ikn}^{4}\left[\delta \left(\omega -\omega_{ikn}\right)-\delta \left(\omega +\omega_{ikn}\right)\right].$$

On the other hand, the average power lost by radiation reaction is readily calculated using Equation (25),

(59) $$\tau {\left(p\cdot \dddot{x}\right)}_{n}=-\frac{2e^{2}}{3c^{3}}\sum_{i}\sum_{k}{\left|x_{ink}\right|}^{2}\omega_{ikn}^{4}.$$

With Equations (58) and (59) introduced into (53) one obtains

(60) $$\frac{d}{dt}{\langle H\rangle }_{n}=-\frac{4e^{2}}{3c^{3}}\sum_{i}\sum_{k}^{\omega_{ink}>0}{\left|x_{ink}\right|}^{2}\omega_{ikn}^{4}.$$

When the particle is in its ground state, there is no net contribution to the sum in Equation (60): the energy lost by radiation is compensated in the mean by the energy extracted from the zpf,and the system remains in the ground state. By contrast, when the particle is in an excited state, both terms are negative for $\omega_{ink}>0$ and contribute to Equation (60). This gives for the rate of change of the energy ([20] and [19] Ch. 6)

(61a) $$\frac{d}{dt}{\langle H\rangle }_{n}=-\sum_{i}\sum_{k}^{\omega_{ink}>0}\hbar \omega_{ink}A_{ink},$$

with

(61b) $$A_{ink}=\frac{4e^{2}}{3\hbar c^{3}}\sum_{i}{\left|x_{ink}\right|}^{2}\omega_{ikn}^{3},$$

which is the qed formula for the Einstein spontaneous emission coefficient. This serves to demonstrate that the radiation reaction and the zpf contribute the same amount to the spontaneous emission rate (see [32] for a discussion of this point in the context of qed). That the result of the sed calculation is in agreement with qed is due to the fact that—in contrast with qm—the zpf is included from the beginning in the description.

As just noted, energy balance is achieved when both particle and field are in their ground state, in which case the terms on the right-hand side of Equation (53) cancel each other,

(62) $$\tau {\langle p\cdot \dddot{x}\rangle }_{o}=-\frac{e}{m}{\langle p\cdot E(t)\rangle }_{o},$$

meaning that the ground state of the particle is absolutely stable in the absence of external radiation. This conclusion applies to any binding potential, in particular to the Coulomb potential. In this connection, it is worth mentioning a series of detailed numerical calculations carried out for the H atom within the sed framework, which have led to the conclusion that the atom immersed in the zpf ionizes eventually, in contradiction with the above results and with experiment; see, e.g., [39,40]. This discrepancy stems from the difference in approaches: in the latter works the calculations are carried out under the assumption that the effect of the zpf on the particle dynamics can be obtained perturbatively. However, a perturbative approach cannot produce qualitative changes of the dynamics, such as those described in this paper. A similar consideration applies to the interesting and elaborate calculational work reported in Ref. [41].

Although the nonperturbative sed approach furnishes a correct description of the quantum regime as a result of the driving effect of the zpf, the detailed *electrodynamical* description of the transition to this regime is an open problem. In this connection, the work carried out in the field of hydrodynamic quantum analogs (hqas) may be illustrative (see [42] and references therein). On one hand, the walking-droplet systems studied in hqa, being macroscopic in nature, lend themselves to direct visualization. On the other hand, several mathematical coincidences have been noted between the hqa and the sed cases, suggesting interesting parallels. One may speculate that, just as has been observed in the hqa case, the nonlinearity of the exact equations for the particle-field system may lead to complex chaotic behavior.

## 7. Final Comments and Conclusions

By recognizing the electromagnetic zpf as an essential part of the quantum ontology, we have addressed several longstanding issues of quantum mechanics. In particular, its presence assigns a physical cause to the quantum fluctuations and thus accounts for the (statistical) indeterministic nature of the quantum phenomenon. The introduction of the zpf is shown to change the (apparently) mechanical nature of the quantum problem into an electrodynamic one, and to induce a drastic, qualitative change in the behavior of an otherwise classical system. Ironically, the zpf itself disappears from the picture at the level of qm, leaving only its ghost behind, in the form of Planck’s constant.

We have come to identify the zpf as a key player. Instead of merely adding the zpf as a recourse to induce a stochastic behavior in an otherwise classical motion, we have placed the emphasis on its deeper role, which is to induce a qualitative change of behavior, leading eventually to qm. Because no perturbative calculation to any order will give rise to a qualitatively different behavior; the new situation entails a shift of approach.

By introducing the zpf into the picture we are considering that atomic matter is made of electric charges (or electromagnetic particles, more generally) that fill the space with radiation as a result of their permanent wiggling. This does not preclude the possibility that other fields should be required at some point to complete the quantum ontology; to arrive at *quantum mechanics,* however, the electromagnetic vacuum proves to be sufficient. In any case, the image of an isolated atom or a quantum particle in empty space appears as an idealization with no real counterpart. If it were experimentally feasible to introduce an atom in a volume completely free of radiation including the zpf, this could provide a valuable possibility to subject the theory to testing. Partial results in this regard were achieved some time ago by introducing atoms in a cavity that modifies the distribution of modes of frequencies of atomic interest, and observing a concomitant effect on the natural decay times of excited states (for early experimental work, see [43,44,45]). As discussed in Section 6, these decay times are determined jointly by the radiation reaction *and* the zpf.

From the perspective gained with the present work, one may say that quantum dynamics is a variant and extension of classical physics into the stochastic domain, which finds a place of its own, both because of the distinctive behavior of quantum systems and for the wealth of phenomena and applications to which it gives rise.

Both authors have made substantial contributions to this paper.
